# Polymorphisms and Circulating Plasma Protein Levels of Immune Checkpoints (CTLA-4 and PD-1) Are Associated With Posner-Schlossman Syndrome in Southern Chinese

**DOI:** 10.3389/fimmu.2021.607966

**Published:** 2021-02-24

**Authors:** Xiaosheng Huang, Xinhua Liu, Ye Ye, Tong Zhang, Shaoyi Mei, Tianhui Zhu, Shiming Peng, Jiamin Cai, Zonghui Yan, Kun Zeng, Danyao Nie, Liangnan Sun, Xiaofeng Hou, Jun Zhao

**Affiliations:** ^1^ Shenzhen Eye Institute, Shenzhen Eye Hospital, Jinan University, Shenzhen, China; ^2^ School of Ophthalmology, Optometry, Shenzhen Eye Hospital, Shenzhen University, Shenzhen, China; ^3^ Department of Ophthalmology, Shenzhen People’s Hospital (The Second Clinical Medical College, Jinan University; The First Affiliated Hospital, Southern University of Science and Technology), Shenzhen, China

**Keywords:** Posner-Schlossman syndrome, CTLA-4, PD-1, genetic variants, soluble molecular

## Abstract

Cytotoxic T-lymphocyte associated protein 4 (CTLA-4) and programmed cell death 1 (PD-1) are well-known key immune checkpoints that play a crucial dampening effect on regulating T-cell homeostasis and self-tolerance. In this study, we aimed to evaluate the association between immune checkpoints (CTLA-4 and PD-1) and Posner-Schlossman syndrome (PSS) in a southern Chinese population. A total of 137 patients with PSS and 139 healthy controls from a southern Chinese population were recruited. Five single nucleotide polymorphisms (SNPs) of *CTLA-4* (rs733618, rs4553808, rs5742909, rs231775, and rs3087243) and five SNPs of *PD-1* (rs10204525, rs2227981, rs2227982, rs41386349, and rs36084323) were genotyped by SNaPshot technique. Soluble CTLA-4 (sCTLA-4) and soluble PD-1 (sPD-1) were determined by ELISA and antibody array assay, respectively. The frequencies of T allele at rs733618 and A allele at rs231775 of *CTLA-4* were significantly higher in PSS patients than in healthy controls (corrected *p* (*P_c_*) = 0.037; *P_c_* = 0.044, respectively). The haplotype frequencies of CACGG haplotype (rs733618-rs4553808-rs5742909-rs231775-rs3087243) of *CTLA-4* and TGAGC haplotype (rs10204525-rs2227981-rs2227982-rs41386349-rs36084323) of *PD-1* in the PSS group was significantly lower than those in the control group (*P_c_* = 0.015, *p* = 0.034, respectively). Circulating plasma levels of sCTLA-4 and sPD-1 in PSS patients were significantly higher than those in controls (all *p* < 0.001). The present study suggests that *CTLA-4* and *PD-1* genetic polymorphisms are associated with the susceptibility to PSS in a southern Chinese population. The upregulated circulating plasma protein levels of sCTLA-4 and sPD-1 might provide some hints regarding the dysfunction of immune checkpoints in PSS during the active status.

## Introduction

Posner-Schlossman syndrome (PSS) is eponymously named after Posner and Schlossman who first described this condition in 1948 (1). PSS is characterized by recurrent unilateral non-granulomas anterior uveitis with elevated intraocular pressure (IOP) (1). The endothelial cell density and progression of retinal nerve fiber layer loss of young adult patients with PSS is caused by frequent attacks of high IOP (2, 3). The mechanism of PSS is still unclear. Many previous studies have reported evidence of cytomegalovirus (CMV) in the aqueous humor and serum (3, 4). Ganciclovir eyedrop (2%) is a novel and effective treatment for PSS patients with high frequent attacks (3). Some previous studies have found that the adaptive immune system was dysfunctional in patients with PSS. Pohlmann et al. found that the expression level of Th1 immune mediator significantly increased in the aqueous humor of patients with PSS (5). Our previous research showed that the Th1- and Th17-related cytokines in the serum might not contribute to PSS (4). Human leukocyte antigen (HLA) allelic heterogenicity might contribute to some differences in PSS prevalence among ethnic populations. Hirose et al. reported that the HLA-Bw54 and HLA-Bw54-Cw1 haplotype were overrepresented in patients with PSS in a Japanese population (6). In previous studies, we found that the HLA-C*14:02 and HLA-E*01:03 alleles, and the HLA-A*11:01-C*14:02, HLA-B*51:01-C*14:02, and HLA-E*01:03-G*01:04 haplotypes confer susceptibility to PSS in a southern Chinese population (7, 8). However, the role of non-HLA genetic variants in PSS still needs to be investigated.

The adaptive immune system maintaining normal function needs appropriate balance between the stimulatory and inhibitory signals (9). The positive costimulatory signal involves the peptide-HLA engagement of the T cell receptor, which is associated with PSS (6–9). The inhibitory signal is caused by the negative immune checkpoints (e.g., CTLA-4 and PD-1) to spare healthy cells and maintain self-tolerance (9). CTLA-4, a close homolog to CD28 and located on chromosome 2q33, competes with CD28 to bind B7.1/7.2 to provide an inhibitory counterbalance at the initial stage of naive T-cell activation (10). PD-1, located on chromosome 2q37, interacts with its ligands to suppress activated T-cell at the later stage of the immune response (9). CTLA-4 and PD-1 contribute to maintaining ocular homeostasis of the immune microenvironment, including ocular immune privilege and anterior chamber-associated immune deviation (11, 12). CTLA-4 and PD-1 are associated with several autoimmune eye diseases. The G allele of rs231775 in *CTLA-4* is associated with Vogt-Koyanagi-Harada (VKH) syndrome and thyroid-associated ophthalmopathy, and the G allele of rs3087243 in *CTLA-4* is associated with scleritis (13–15). The G/G genotype of rs10204525 in *PD-1* is associated with acute anterior uveitis, and the G/G genotype of rs2227981 in *PD-1* is associated with sympathetic ophthalmia and the occurrence of extraocular manifestations of VKH syndrome (16, 17). Splenic CD4^+^ T cells expressing CTLA-4 and PD-1 contributed to the induction of anterior chamber-associated immune deviation (11, 12). High expression of PD-1 mitigated inflammation during the active phase of experimental autoimmune uveitis mouse model (18). PD-1 is highly expressed in the inflammation sites of herpes simplex, keratitis, autoimmune uveitis, diabetic retinopathy, and thyroid-associated ophthalmopathy (19). Thus, CTLA-4 and PD-1 might contribute to the progression of some eye diseases.

However, the association between the immune checkpoints (CTLA-4 and PD-1) and PSS is unclear. In the present study, we investigated 10 SNPs of CTLA-4 and PD-1 and quantitatively assessed soluble CTLA-4 (sCTLA-4) and soluble PD-1 (sPD-1) from circulating plasma in a southern Chinese population with PSS. We aimed to evaluate whether genetic heterogeneity of immune checkpoints (CTLA-4 and PD-1) and their protein expression levels contribute to PSS during the active status.

## Materials and Methods

### Study Participants

A total of 137 patients with PSS attending the Clinic of Shenzhen Eye Hospital were included in the study between January 2018 and March 2020. Patients were diagnosed with PSS according to the following classical criteria and amendments (1, 3, 4): i) recurrent attacks of mild discomfort in one eye; ii) elevated IOP > 21 mmHg with duration of attack. The IOP may reach 50 mmHg without peripheral anterior synechia; iii) a few white mutton-fat keratic precipitates; iv) no significant decrease or slight decrease in visual acuity, no visual field loss, and optic nerve damage in patients with shorter course of disease; and v) no history of other eye diseases except for refractive error. All patients were in the active disease phase with either a first attack or in the early stage of a recurrent one. One hundred and thirty-nine unrelated subjects were recruited at the Shenzhen Blood Center from healthy volunteer blood donors without eye disease. Patients and controls were all southern Han Chinese and matched for age, sex, and ethnicity. Written informed consent was obtained from all study participants.

### Specimen Collection

The peripheral blood of all participants was collected in EDTA vial. Plasma was separated by centrifugation (3,000×g, 20 min, 4°C) within 2 h after blood collection. The peripheral venous blood and plasma were stored at −80°C until further analysis.

### DNA Extraction, Polymerase Chain Reaction Amplification, and Genotyping

The SNPs, that we selected for genotyping in this study could potentially regulate the function and expression of these genes based on previous studies in **Supplementary Table 1** (13, 15–17). Genomic DNA was extracted from peripheral blood samples of all participants using the TIANamp Blood DNA Kit (TIANGEN Biotech, Beijing, China). PCR amplification was performed in a 20 μl reaction system, including 1 μl genomic DNA, 0.5 μl of each PCR primer in a total volume of 20 μl containing 1× HotStarTaq buffer, 2.0 mM Mg^2+^, 0.3 mM dNTP, and 1 U HotStarTaq polymerase (Qiagen, Hilden, Germany). The cycling conditions for *CTLA-4* and *PD-1* were 95°C for 2 min; followed by 35 cycles at 96°C for 20 s, 62°C; for 2 min, and 72°C for 3 min; and a final extension at 72°C for 10 min. The amplified samples were maintained at 4°C. SNaPshot multiplex single-base extension reaction primer sequences of each SNP are listed in **Supplementary Table 2**. The extension reaction was performed in a 10 μl reaction system, including 2 μl purified PCR product, 1 μl primer (final concentration: 0.8 mM), 5 μl SNaPshot Multiplex Kit (Applied Biosystems, Foster City, CA, USA), and 2 μl ultrapure water. The cycling conditions for extension for *CTLA-4* and *PD-1* were 96°C for 1 min; followed by 28 cycles at 96°C for 10 s, 52°C for 5 s, and 60°C for 30 s; with a final hold at 4°C. Finally, 10 μl of the extension product was purified with 1 U SAP for 1 h at 37°C and inactivated for 15 min at 75°C. The resulting data were analyzed with an ABI3130XL sequencer and GeneMapper version 4.0 Software (Applied Biosystems, Co. Ltd., USA).

### Determination of Plasma Protein Levels of sCTLA-4 and sPD-1 by Double Antibody Sandwich ELISA and Confirmed by Antibody Array Assay

Commercially available ELISA kits were used to measure the concentrations of sCTLA-4 and sPD-1 (Jianglai Bio, Shanghai, China) according to the manufacturer’s instructions. The assay sensitivities for sCTLA-4 and sPD-1 were 0.1 and 1.0 pg/ml, respectively. The range of detection was 0.25–8 ng/ml and 7.5–240 pg/ml for sCTLA-4 and sPD-1, respectively. Plasma samples were added into the microplate wells and incubated at 37°C for 30 min according to the manufacturer’s instructions (Jianglai Bio, Shanghai, China). The horseradish peroxidase-labeled antibody was then added and incubated at 37°C for 30 min. TMB (3,3’,5,5’-tetramethylbenzidine) solution was added to each well and incubated for 15 min for the color reaction. The reaction was terminated by adding stop solution. The absorbance (A) at 450 nm was measured using a microplate reader (Model 680, Bio-Rad Laboratories Inc., Japan). The plasma levels of sCTLA-4 and sPD-1 were calculated based on the A values of the samples.

The plasma protein levels of sCTLA-4 and sPD-1 were contained using the human immune checkpoint molecule array (RayBiotech, USA, QAH-ICM-1-1) according to the manufacturer’s instructions, as described by Chang et al. (20).

### Detection of CMV-IgG and CMV-IgM Antibodies by Indirect ELISA and Chemiluminescent Immunoassay

The CMV-IgG and -IgM antibodies were determined in the plasma of a subset of samples including 50 patients with PSS and 54 heathy controls by indirect ELISA (Virion/Serion, Germany) and chemiluminescent immunoassay (LIASON^®^, DiaSorin, Italy) according to the manufacturer’s recommendations.

### Statistical Analysis

Statistical analysis was performed using SPSS (version 22.0, SPSS Inc., Chicago, IL, USA). Age and IOP were compared between patients with PSS and controls, using independent-samples *t*-test. Hardy-Weinberg equilibrium (HWE) was evaluated using the chi-squared test. The frequencies of alleles and genotypes were obtained through direct counting. We used using PLINK (ver 1.07, http://pngu.mgh.harvard.edu/purcell/plink/) to construct haplotypes and estimate haplotype frequencies for both cases and controls (21). Linkage disequilibrium (LD) and haplotype blocks were estimated using the Haploview 4.2 program (22). Since sCTLA-4 and sPD-1 were not consistent with normal distribution as suggested by the Shapiro-Wilk test, the data were presented as median and interquartile range (Q1, Q3), and Mann-Whitney U test was used to compare the differences between the two groups. The differences in sex, allele frequency, genotype frequency, and haplotype frequency between cases and controls were evaluated using the chi-squared test or Fisher’s exact test. We used Benjamin & Hochberg step-up false discovery rate (FDR) to correct multiple testing. The value of *p* < 0.05 was considered to indicate statistical significance. Odds ratio (OR) and 95% confidence interval (CI) were calculated whenever applicable.

## Results

### Demographic and Clinical Features of Study Participants

The mean age of the PSS group [72 males (52.5%) and 65 females (47.5%)] was 39.6±12.5 years. The mean age of the control group [74 males (53.2%) and 65 females (46.8%)] was 41.3±10.4 years. No significant intergroup differences were found with respect to age and sex (*p* = 0.89 and 0.32, respectively, **Table 1**). The mean IOP of the PSS group was 40.9±5.7 mmHg, which was significantly higher than that of the controls (15.3±3.2 mmHg) (*p* < 0.001, **Table 1**).

**Table 1:** The demographic and clinical features of the patients with PSS and controls.

Feature	PSS (*n* = 137)	Control (*n* = 139)	*p*
Sex (M/F)	72/65	74/65	0.89^a^
Age (year, mean ± SD)	39.6±12.5	41.3±10.4	0.32^b^
IOP (mmHg, mean ± SD)	40.9±5.7	15.3±3.2	<0.001^b^
KPs (Y/N)	Y	N	

^a^Chi-squared test; ^b^independent-samples t-test; PSS, Posner-Schlossman syndrome; M, male, F, female; IOP, intraocular pressure; KPs, keratic precipitates; Y, yes; N, no.

### *CTLA-4* Genotype and Allele Frequencies

Genotype and allele frequencies of *CTLA-4* rs733618, rs4553808, rs5742909, rs231775, and rs3087243 are depicted in **Table 2**. The genotype distributions of *CTLA-4* SNPs in control groups did not violate the HWE (all *p* > 0.05). The frequencies of T/T genotype at rs733618 and the A/A genotype at rs231775 and rs3087243 of *CTLA-4* were significantly higher in PSS patients than in healthy controls, but did not survive the FDR correction (51.82 *vs.* 35.52%, *p* = 0.005, corrected p (*P_c_*) = 0.082, OR = 1.81; 21.90 *vs.* 9.35%, *p* = 0.004, *P_c_* = 0.122, OR = 2.72; 8.76 *vs.* 2.88%, *p* = 0.037, *P_c_* = 0.37, OR = 3.24, respectively). The frequencies of T allele at rs733618 and A allele at rs231775 of *CTLA-4* were significantly higher in PSS patients than in healthy controls, and these associations survived the FDR correction (70.80 *vs.* 58.99%, *P_c_* = 0.037, OR = 1.56; 40.88 *vs.* 30.22%, *P_c_* = 0.044, OR = 1.55, respectively). Our results showed that there were no significant differences in the genotype and allele frequencies of rs4553808 and rs5742909 in *CTLA-4* between PSS patients and controls (all *p* > 0.05). The raw genotype data of the samples was shown in the **Supplementary Table 3**.

**Table 2:** Genotype and allele frequencies of *CTLA-4* SNPs in the PSS patients and healthy controls.

	PSS *n* = 137 (%)	Control *n* = 139 (%)	*p*	*P*_c_	OR	95% CI
**rs733618**						
Genotype						
**TT**	**71 (51.82)**	**49 (35.25)**	**0.005**	0.082	**1.81**	**1.12−2.92**
CT	52 (37.96)	66 (47.48)	0.110	0.550	0.68	0.42−1.10
CC	14 (10.22)	24 (17.27)				
Allele						
**T**	**194 (70.80)**	**164 (58.99)**	**0.004**	**0.037**	**1.56**	**1.11−2.21**
C	80 (29.20)	114 (41.01)				
rs4553808						
Genotype						
AA	103 (75.18)	112 (80.58)	0.280	0.560	0.73	0.41−1.29
AG	28 (20.44)	25 (17.99)	0.605	0.726	1.17	0.67−2.13
GG	6 (4.38)	2 (1.44)				
Allele						
A	234 (85.40)	249 (89.57)	0.139	0.278	0.68	0.41−1.12
G	40 (14.60)	29 (10.43)				
rs5742909						
Genotype						
CC	104 (75.91)	113 (81.29)	0.275	0.635	0.71	0.40−1.26
CT	27 (19.71)	24 (17.27)	0.605	0.698	1.22	0.67−2.22
TT	6 (4.38)	2 (1.44)				
Allele						
C	235 (85.44)	250 (89.93)	0.134	0.336	0.67	0.40−1.12
T	39 (14.23)	28 (10.07)				
**rs231775**						
Genotype						
**AA**	**30 (21.90)**	**13 (9.35)**	**0.004**	0.122	**2.72**	**1.35−5.47**
AG	52 (37.96)	58 (41.73)	0.522	0.746	0.87	0.53−1.38
GG	55 (40.15)	68 (48.92)				
Allele						
**A**	**112 (40.88)**	**84 (30.22)**	**0.009**	**0.044**	**1.553**	**1.10−2.20**
G	162 (59.12)	194 (69.78)				
**rs3087243**						
Genotype						
**AA**	**12 (8.76)**	**4 (2.88)**	**0.037**	0.370	**3.24**	**1.028−10.31**
AG	48 (35.04)	48 (34.53)	0.930	0.930	1.02	0.62−1.68
GG	77 (56.20)	87 (62.59)				
Allele						
A	72 (26.28)	56 (20.14)	0.088	0.293	1.36	0.92−2.02
G	202 (73.72)	222 (79.86)				

Values in bold indicate significant differences. p value was calculated using χ2 test or Fisher’s exact test and corrected for multiple testing using the FDR method. PSS, Posner-Schlossman syndrome; P_c_, corrected p value; OR, odds ratio; CI, confidence interval.

### *PD-1* Genotype and Allele Frequencies

Genotype and allele frequencies of *PD-1* rs10204525, rs2227981, rs2227982, rs41386349, and rs36084323 are depicted in **Table 3**. The genotype distributions of *PD-1* SNPs in control groups did not violate HWE (all *p* > 0.05). Our results showed that there were no significant differences in the genotype and allele frequencies of rs10204525, rs2227981, rs2227982, rs41386349, and rs36084323 in *PD-1* between PSS patients and controls (all *p* > 0.05). The raw genotype data of the samples was shown in the **Supplementary Table 3**.

**Table 3:** Genotype and allele frequencies of *PD-1* SNPs in the PSS patients and healthy controls.

	PSS *n* = 137 (%)	Control *n* = 139 (%)	*p*	*P*_c_	OR	95% CI
rs10204525						
Genotype						
TT	70 (52.45)	83 (59.71)	0.150	0.500	0.71	0.44−1.14
CT	59 (41.96)	50 (35.97)	0.228	0.570	1.35	0.83−2.19
CC	8 (5.59)	6 (4.32)				
Allele						
T	199 (72.63)	216 (77.70)	0.168	0.240	0.76	0.52−1.12
C	75 (27.37)	62 (22.30)				
rs2227981						
Genotype						
AA	17 (12.41)	9 (6.47)	0.092	0.552	2.05	0.88−4.77
AG	45 (32.85)	52 (37.41)	0.427	0.641	0.82	0.50−1.34
GG	75 (54.74)	78 (56.21)				
Allele						
A	79 (28.83)	70 (25.18)	0.334	0.417	1.20	0.83−1.75
G	195 (71.17)	208 (74.82)				
rs2227982						
Genotype						
AA	40 (29.20)	47 (33.81)	0.409	0.646	0.81	0.49−1.34
AG	60 (43.80)	65 (46.76)	0.621	0.690	0.89	0.55−1.43
GG	37 (27.01)	27 (19.42)				
Allele						
A	140 (51.09)	159 (57.19)	0.150	0.251	0.78	0.56−1.09
G	134 (48.91)	119 (42.81)				
rs41386349						
Genotype						
AA	10 (7.30)	6 (4.32)	0.289	0.510	1.75	6.62−4.94
AG	39 (28.47)	48 (34.78)	0.278	0.596	0.75	0.45−1.26
GG	88 (64.23)	85 (61.15)				
Allele						
A	59 (21.53)	58 (21.01)	0.882	0.882	1.03	0.69−1.55
G	215 (78.47)	218 (78.99)				
rs36084323						
Genotype						
CC	36 (26.28)	30 (21.58)	0.361	0.602	1.30	0.74−2.26
CT	62 (45.26)	65 (46.76)	0.802	0.859	0.94	0.59−1.51
TT	39 (28.47)	44 (31.65)				
Allele						
C	134 (48.91)	125 (44.96)	0.354	0.553	1.17	0.84−1.64
T	140 (51.09)	153 (55.04)				

The p value was calculated using χ2 test or Fisher’s exact test and corrected for multiple testing using the FDR method. PSS, Posner-Schlossman syndrome; P_c_, corrected p value; OR, odds ratio; CI, confidence interval.

### *CTLA-4* and *PD-1* Haplotype Frequencies and Linkage Disequilibrium

Four haplotypes of *CTLA-4* and six haplotypes of *PD-1* were detected (**Table 4**). The haplotype frequency of CACGG haplotype (rs733618-rs4553808-rs5742909-rs231775-rs3087243) of *CTLA-4* in the PSS group was significantly lower than that in the control group (29.30 *vs.* 41.16%, *P_c_* = 0.015, OR = 0.60). The haplotype frequency of TGAGC haplotype (rs10204525-rs2227981-rs2227982-rs41386349-rs36084323) of *PD-1* in the PSS group was significantly lower than that in the control group (0.73 *vs.* 3.27%, *p* = 0.034, OR = 0.22), although this association did not survive the FDR correction (*P_c_* = 0.204). No significant difference in the other haplotypes was found between the two groups (all *p* > 0.05).

**Table 4:** *CTLA-4* and *PD-1* haplotype frequencies of the PSS patients and healthy controls.

	Frequency (%)		*p*	*P*_c_	OR	95% CI
	PSS *2n* = 274	Control *2n* = 278				
CTLA-4 rs733618-rs4553808-rs5742909-rs231775-rs3087243						
*TACAA*	26.37	19.86	0.070	0.139	1.45	0.97−2.15
*TGTAG*	14.29	10.11	0.134	0.179	1.48	0.88−2.49
***CACGG***	**29.30**	**41.16**	**0.004**	**0.015**	**0.60**	**0.42−0.85**
*TACGG*	30.04	28.88	0.767	0.766	1.06	0.73−1.53
PD-1 rs10204525-rs2227981-rs2227982-rs41386349-rs36084323						
TAGAC	20.80	19.11	0.639	0.639	1.11	0.73−1.68
*CAGGC*	7.30	4.52	0.203	0.609	1.61	0.77−3.35
*CGGGC*	20.07	17.46	0.420	0.630	1.19	0.78−1.83
***TGAGC***	**0.73**	**3.27**	**0.034**	0.204	**0.22**	**0.05−1.02**
TAGAT	0.73	1.82	0.450	0.540	0.40	0.08−2.07
*TGAGT*	50.36	53.82	0.395	0.790	0.87	0.62−1.21

Values in bold indicate significant differences. The p value was calculated using χ2 test or Fisher’s exact test and corrected for multiple testing using the FDR method. PSS, Posner-Schlossman syndrome; P_c_: corrected p value; OR, odds ratio; CI, confidence interval.

**Figure 1:**
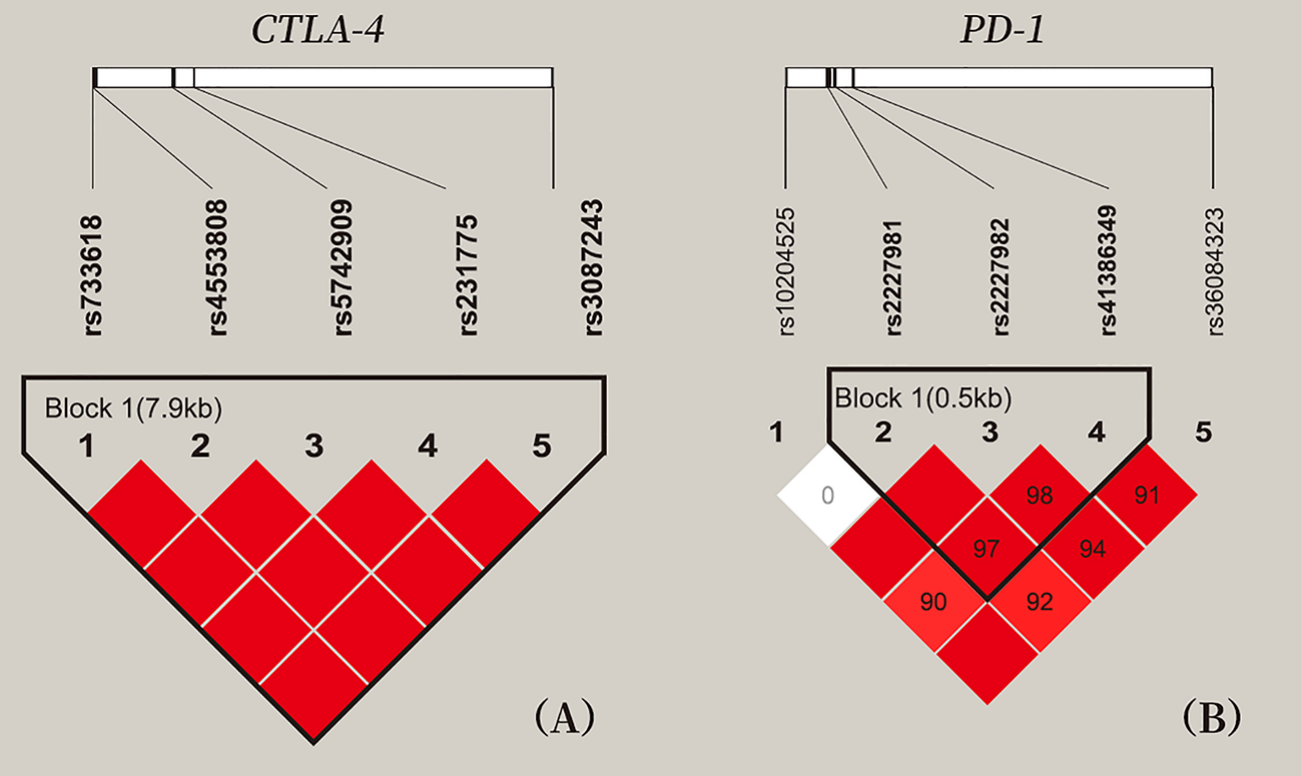
Location and pair-wise linkage disequilibrium values of CTLA-4 and PD-1 SNPs in all study participants. **(A)** These five SNPs span 7 kb of *CTLA-4* gene region. **(B)** These five SNPs of *PD-1* gene region. Values of the pair-wise D' (×100) are shown in blocks (D' values of 1.0 are not presented). Haplotype blocks were estimated with the Haploview program.

LD analysis among five SNPs of *CTLA-4* and five SNPs of *PD-1* showed that all pair-wise LD between *CTLA-4* SNPs and *PD-1* SNPs had strong LD (D’ > 0.9), expect for the weak LD between rs10204525 and rs2227981 (D’ = 0; **Figure 1**).

### Circulating Plasma Protein Levels of sCTLA-4 and sPD-1

**Figure 2:**
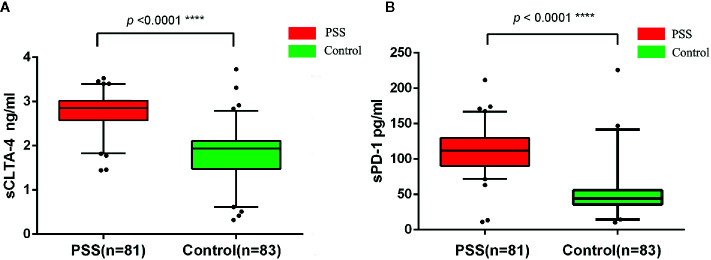
Expression anaysis of sCTLA-4 and sPD-1 by ELISA. **(A)** Comparison of circulating plasma protein levels of sCTLA-4 between PSS patients and controls. **(B)** Comparison of circulating plasma protein levels of sPD-1 between PSS patients and controls. PSS: Posner-Schlossman syndrome; sCTLA-4: soluble Cytotoxic T-lymphocyte associated protein 4; sPD-1: soluble programmed cell death 1.

The circulating plasma protein levels of sCTLA-4 and sPD-1 in PSS patients (n = 81) were significantly higher than those in controls (n = 83) by ELISA (2.76±0.43 *vs.* 1.78±0.63 ng/ml, *p* < 0.0001; 110.31±30.60 *vs.* 47.98±27.72 pg/ml, *p* < 0.0001, respectively; **Figure 2**). Our study did not find any significant difference in plasma protein levels of sCTLA-4 and sPD-1 according to the genotypes of *CTLA-4* and *PD-1* (all *p* > 0.05; data not shown).

**Figure 3:**
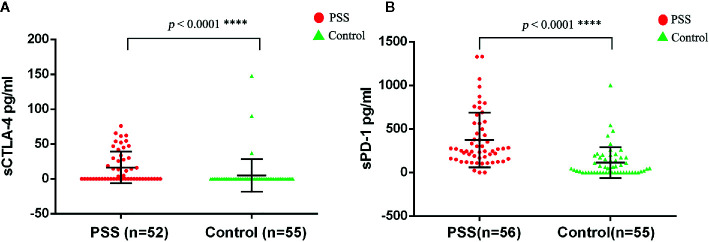
Expression anaysis of sCTLA-4 and sPD-1 by antibody array assay. **(A)** Comparison of circulating plasma protein levels of sCTLA-4 between PSS patients and controls. **(B)** Comparison of circulating plasma protein levels of sPD-1 between PSS patients and controls. PSS: Posner-Schlossman syndrome; sCTLA-4: soluble Cytotoxic T-lymphocyte associated protein 4; sPD-1: soluble programmed cell death 1.

The circulating plasma protein levels of sCTLA-4 and sPD-1 were confirmed by the human immune checkpoint molecular array. The circulating plasma protein levels of sCTLA-4 in PSS patients (n = 52) were significantly higher than those in controls (n = 55) (16.37±22.73 *vs.* 4.99±23.53 pg/ml, *p* < 0.0001; **Figure 3A**). The circulating plasma protein levels of sPD-1 in PSS patients (n = 56) were significantly higher than those in controls (n = 55) (373.35±314.27 *vs.* 112.40±176.20 pg/ml, *p* < 0.0001; **Figure 3B**).

### Comparison of *CTLA-4* and *PD-1* Allele Frequencies and Levels of sCTLA-4 and sPD-1 Between CMV-IgG (+) PSS Patients and CMV-IgG (+) Controls

A subset of samples including 50 patients with PSS and 54 heathy controls were available for detection of the CMV antibodies. None of these samples was positive for CMV-IgM, and all these samples were positive for CMV-IgG. The frequencies of T allele of rs733618, A allele of rs231775, and A allele of rs3087243 in *CTLA-4* were significantly higher in CMV-IgG (+) PSS patients than in CMV-IgG (+) controls, although these associations did not survive the correction for multiple testings (71.0 *vs.* 55.6%, *p* = 0.021, *P_c_* = 0.21, OR=1.96; 46.0 *vs.* 29.6%, *p* = 0.015, *P_c_* = 0.075, OR=2.02; 32.0 *vs.* 19.4%, *p* = 0.038, *P_c_* = 0.13, OR=1.95, respectively; **Table 5**). No significant difference was found in allele frequencies of rs10204525, rs2227981, rs2227982, rs41386349, and rs36084323 in *PD-1* between CMV-IgG (+) PSS patients and CMV-IgG (+) controls (all *p* > 0.05). Circulating plasma levels of sCTLA-4 and sPD-1 in CMV-IgG (+) PSS patients were significantly higher than those in controls (*p* < 0.0001; **Table 6**).

**Table 5:** Allele frequencies of *CTLA-4* and *PD-1* SNPs in the CMV IgG (+) PSS patients and healthy controls.

	CMV IgG (+) PSS *n* = 50 (%)	CMV IgG (+) control *n* = 54 (%)	*p*	*P_c_*	OR	95% CI
*CTLA-4*						
**rs733618**						
**T**	**71 (71.00)**	**60 (55.56)**	**0.021**	0.210	**1.959**	**1.11−2.21**
C	29 (29.00)	48 (44.44)				
rs4553808						
A	86 (86.00)	96 (88.89)	0.529	0.756	0.768	0.34−1.75
G	14 (14.00)	12 (11.11)				
rs5742909						
T	14 (14.00)	11 (10.19)	0.398	0.796	1.436	0.62−3.33
C	86 (86.00)	97 (89.81)				
**rs231775**						
**A**	**46 (46.00)**	**32 (29.63)**	**0.015**	0.075	**2.023**	**1.14−3.58**
G	54 (54.00)	76 (70.37)				
**rs3087243**						
**A**	**32 (32.00)**	**21 (19.44)**	**0.038**	0.127	**1.95**	**1.03−3.68**
G	68 (68.00)	87 (80.56)				
*PD-1*						
rs10204525						
T	73 (73.00)	83 (76.85)	0.522	0.870	0.814	0.43−1.53
C	27 (27.00)	25 (23.15)				
rs2227981						
A	27 (27.00)	32 (29.63)	0.674	0.843	0.878	0.48−1.61
G	73 (73.00)	76 (70.37)				
rs2227982						
A	53 (53.00)	56 (51.85)	0.868	0.868	1.047	0.61−1.81
G	47 (47.00)	52 (48.15)				
rs41386349						
A	20 (20.00)	27 (25.00)	0.389	0.973	0.750	0.39−1.45
G	80 (80.00)	81 (75.00)				
rs36084323						
T	53 (53.00)	55 (50.93)	0.765	0.850	1.087	0.63−1.87
C	47 (47.00)	53 (49.07)				

Values in bold indicate significant differences. The p value was calculated using χ2 test or Fisher’s exact test and corrected for multiple testing using the FDR method. PSS, Posner-Schlossman syndrome; CMV, cytomegalovirus; P_c_, corrected p value; OR, odds ratio; CI, confidence interval.

**Table 6:** Comparison of plasma levels of sCTLA-4 and sPD-1 between CMV IgG (+) PSS patients and healthy controls.

	*n*	sCTLA-4 (ng/ml)	sPD-1 (pg/ml)
CMV IgG (+) PSS	50	2.75±0.46	108.85±29.57
CMV IgG (+) Control	54	1.77±0.63	49.01±32.54
Z		6.848	7.664
*p*		**<0.0001**	**<0.0001**

Values in bold indicate significant differences. The p value was calculated using Mann-Whitney U test. PSS, Posner-Schlossman syndrome; CMV, cytomegalovirus.

## Discussion

Immune checkpoints prevent the excessive immune response by transferring the inhibitory signals to spare healthy cells and maintain self-tolerance (9). CTLA-4 and PD-1, the most widely investigated checkpoints, are reportedly upregulated by the tumor to escape from immune system monitoring (10). The HLA polymorphism confers susceptibility to PSS. However, little is known about the non-HLA genetic background of PSS (3).

In the present study, we genotyped five *CTLA-4* SNPs and five *PD-1* SNPs in 137 patients with PSS and 139 healthy controls from a southern Chinese population. The frequencies of T allele of rs733618 and A allele of rs231775 in *CTLA-4* were significantly higher in PSS patients than in controls, and these associations survived multiple testings correction. The frequencies of T/T genotype of rs733618 and A/A genotype of rs231775 and rs3087243 in *CTLA-4* were significantly higher in PSS patients than in controls, but did not survive the FDR correction. The CACGG haplotype (rs733618-rs4553808-rs5742909-rs231775-rs3087243) in *CTLA-4* might be the protective haplotype for PSS. Du et al. reported that the G allele at rs231775 and ACGG at *CTLA-4* rs4553808-rs5742909-rs231775-rs3087243 might be risk factors for VKH syndrome (13). Since the risk genotype of rs733618 and rs231775, and the haplotype of rs4553808-rs5742909-rs231775-rs3087243 of *CTLA-4* in PSS were completely different from those in VKH syndrome, we considered that *CTLA-4* genetic background might be different between PSS and VKH syndrome (13). SNP rs231775 of the *CTLA*-*4* gene causes adenine-guanine dimorphism at codon 17 in the peptide leader sequence (23). The A and G alleles of rs231775 encodes the CTLA-4 ^17^Thr and CTLA-4 ^17^Ala, respectively. Sun et al. reported that the CTLA-4 ^17^Thr enhances the interaction between CTLA-4 and B7.1 molecule, and carries greater inhibitory effects on the proliferation and activation process of CD25^+^/CD4^+^ T cell in PBMCs treated with or without phytohemagglutinin, than the CTLA-4^17^ Ala (23, 24). SNP rs733618 is located upstream of the *CTLA-4* gene and is a part of the gene’s activator sequence. The substitution of C allele to T allele of rs733618 could affect the gene expression by changing the sequence of transcription factor regulated NF-1 and c/EBPβ binding sites (25). We hypothesized that the A allele of rs231775 and T allele of rs733618 could confer susceptibility to PSS in the southern Chinese population by impacting the expression and function of *CTLA-4*. The circulating plasma protein levels of sCTLA-4 were increased during the onset of disease in PSS patients compared with healthy controls, which also occurs in other autoimmune diseases (e.g., autoimmune thyroid disease, Graves’ ophthalmopathy, myasthenia gravis, and spondyloarthropathies) (26–28). The relevance of sCTLA-4, primarily secreted by Treg cell, to immune regulation remains controversial. The sCTLA-4 maintains the ability to bind to the B7 ligand *via* the MYPPPY motif on antigen presenting cells, and prevents the B7 ligand from combining with CD28 to inhibit T cell responses (29). Furthermore, the selective blockade of sCTLA-4 activates the proliferation of T cells and upregulated the expression of Th1/Th17-related cytokines (30). The significant antigen-specific immune responses were increased by using the anti-sCTLA-4-mAb *in vitro* and *in vivo* (30). However, we did not find significant association between the genotype and expression of sCTLA-4 (data not shown), probably because we only investigated a limited number of SNPs in this study. More related SNPs, the transcriptional regulation and protein modification of CTLA-4 in the onset of PSS are needed to be investigated in the future. We concluded that the A allele of rs231775 and T allele of rs733618 were risk factors for PSS, and the expression of sCTLA-4 increased the participation of T-cell regulation in the B7/CTLA-4/CD28 signaling pathway during the onset of PSS.

With regard to another important immune checkpoint, PD-1, we found that the polymorphisms of *PD-1* were not associated with PSS, except that the TGAGC haplotype (rs10204525-rs2227981-rs2227982-rs41386349-rs36084323) of *PD-1* was lower in patients with PSS than in the controls. Interestingly, the expression of sPD-1 increased during the onset of PSS compared to controls. High expression of sPD-1 might contribute to the process of PSS through transforming the PD-1/PD-L1 signaling pathway or regulating the immune response of the T cell. In some chronic viral infections, higher PD-1 likely converts effector cytotoxic T lymphocytes into exhausted cytotoxic T lymphocytes (e.g., human immunodeficiency virus, hepatitis B virus, hepatitis C virus) (31). High PD-1 expression is associated with emerging and evident CMV disease and with viremia in liver transplant recipients (32). PD-1 was also associated with the viremia caused by chronic CMV infection, and blocking PD-1 signaling might help recover the function of exhausted T cells in chronic CMV infection (33). High expression of PD-1 is related to CMV infection, which might be the potential crucial factor for PSS (3). The sPD-1 enhanced the immune response by enhancing co-delivered antigen-specific CD8^+^ T-cell responses and *in vivo* maturation of DCs during activation of naive CD8^+^ T cells, and inhibited the PD-1/PD-L1 interaction (34, 35). However, the mechanisms of upregulated expression of sPD-1 in the onset of PSS require future investigation.

Since the CMV infection might be an important risk factor for PSS, we analyzed the associations of the *CTLA-4* and *PD-1* SNPs and the expression levels of sCTLA-4 and sPD-1 with PSS in the CMV-IgG (+) samples. Our results showed that rs733618, rs231775, and rs3087243 in *CTLA-4* were significantly associated with PSS in the CMV-IgG (+) individuals, although these associations did not survive the FDR correction, probably due to the limited sample sizes available for detection of the CMV antibodies (**Table 5**). The circulating plasma protein levels of sCTLA-4 and sPD-1 were increased during the onset of disease in CMV-IgG (+) PSS patients compared with CMV-IgG (+) healthy controls (**Table 6**). Our study did not find any significant difference when plasma protein levels of sCTLA-4 and sPD-1 were analyzed according to the genotypes of CTLA-4 and PD-1 (all *p* > 0.05). We hypothesize that the mechanisms of increased expression of sCTLA-4 and sPD-1 might be complex in the onset of PSS. Moreover, the transcriptional regulation and protein modification of sCTLA-4 and sPD-1 worth to be investigated in the onset of PSS.

In summary, the results of the present study suggest that the T allele of rs733618 and the A allele of rs231775 in *CTLA-4* might contribute to the process of PSS in a southern Chinese population. The CACGG haplotype (rs733618-rs4553808-rs5742909-rs231775-rs3087243) of *CTLA-4* and TGAGC haplotype (rs10204525-rs2227981-rs2227982-rs41386349-rs36084323) of *PD-1* might be protective factors against PSS in southern Chinese patients. Moreover, sCTLA-4 and sPD-1 were increased in the onset of PSS. These findings suggest that the polymorphism and soluble levels of *CTLA-4* and *PD-1* might shed further light on the dysfunction of immune checkpoints in PSS.

## Data Availability Statement

The raw data was shown in the **Supplementary Table 3**.

## Ethics Statement

The studies involving human participants were reviewed and approved by the local Ethics Committee of Shenzhen Eye Hospital. The patients/participants provided their written informed consent to participate in this study.

## Author Contributions

XHu, XL, and JZ conceived and designed the experiments. XHu, YY, TZ, XHo, and JZ performed the experiments and analyzed the data. XHu, XL, YY, SM, SP, JC, ZY, KZ, DN, LS, and JZ contributed to patients and control recruitments. XHu and JZ wrote the paper. All authors contributed to the article and approved the submitted version.

## Funding

This study was supported by the Science, Technology, and Innovation Commission of Shenzhen Municipality under Grant (number JCYJ20180228164400218 and GJHZ20180420180937076) and Sanming Project of Medicine in Shenzhen Grant (number SZSM201812090).

## Conflict of Interest

The authors declare that the research was conducted in the absence of any commercial or financial relationships that could be construed as a potential conflict of interest.

## References

[1] Posner A Schlossman A. Syndrome of unilateral recurrent attacks of glaucoma with cyclitic symptoms. Arch Ophthalmol (1948) 39(4):517–35. 10.1001/archopht.1948.00900020525007 18123283

[2] Maruyama K Maruyama Y Sugita S Mori K Yokoyama Y Sanuki-Kunimatsu S. Characteristics of cases needing advanced treatment for intractable Posner-Schlossman syndrome. BMC Ophthalmol (2017) 17(1):45. 10.1186/s12886-017-0438-y 28399831 PMC5387341

[3] Megaw R Agarwal PK. Posner-Schlossman syndrome. Surv Ophthalmol (2017) 62(3):277–85. 10.1016/j.survophthal.2016.12.005 28012873

[4] Zhao J Chen W Huang X Peng S Zhu T Deng Z. Serum Th1 and Th17 related cytokines and autoantibodies in patients with Posner-Schlossman syndrome. PloS One (2017) 12(4):e0175519. 10.1371/journal.pone.0175519 28384257 PMC5383301

[5] Pohlmann D Schlickeiser S Metzner S Lenglinger M Winterhalter S Pleyer U. Different composition of intraocular immune mediators in Posner-Schlossman-Syndrome and Fuchs’ Uveitis. PloS One (2018) 13(6):e0199301. 10.1371/journal.pone.0199301 29944680 PMC6019249

[6] Hirose S Ohno S Matsuda H. HLA-Bw54 and glaucomatocyclitic crisis. Arch Ophthalmol (1985) 103(12):1837–9. 10.1001/archopht.1985.01050120071023 4074175

[7] Zhao J Zhu T Chen W Fan BJ He L Yang B. Human leukocyte antigens-B and -C loci associated with Posner-Schlossman syndrome in a southern Chinese population. PloS One (2015) 10(7):e0132179. 10.1371/journal.pone.0132179 26161794 PMC4498812

[8] Huang X Xu Y Chen W Zhu T He L Wang S. The genetic contribution of HLA-E*01:03 and HLA-E*01:03-G*01:01 to Posner-Schlossman syndrome in southern Chinese. Ann Trans Med (2019) 7(23):749. 10.21037/atm.2019.11.70 PMC6989981 32042765

[9] Sharpe AH Pauken KE. The diverse functions of the PD1 inhibitory pathway. Nat Rev Immunol (2018) 18(3):153–67. 10.1038/nri.2017.108 28990585

[10] Gu D Ao X Yang Y Chen Z Xu X. Soluble immune checkpoints in cancer: production, function and biological significance. J Immunother Cancer (2018) 6(1):132. 10.1186/s40425-018-0449-0 30482248 PMC6260693

[11] Zhu X Yang P Zhou H Li B Huang X Meng Q. CD4+CD25+Tregs express an increased LAG-3 and CTLA-4 in anterior chamber-associated immune deviation. Graefe’s Arch Clin Exp Ophthalmol (2007) 245(10):1549–57. 10.1007/s00417-007-0591-8 17541623

[12] Meng Q Yang P Li B Zhou H Huang X Zhu L. CD4+PD-1+ T cells acting as regulatory cells during the induction of anterior chamber-associated immune deviation. Invest Ophthalmol Visual Sci (2006) 47(10):4444–52. 10.1167/iovs.06-0201 17003438

[13] Du L Yang P Hou S Lin X Zhou H Huang X. Association of the CTLA-4 gene with Vogt-Koyanagi-Harada syndrome. Clin Immunol (2008) 127(1):43–8. 10.1016/j.clim.2008.01.004 18282809

[14] Wang H Zhu LS Cheng JW Cai JP Li Y Ma XY. Meta-analysis of association between the +49A/G polymorphism of cytotoxic T-lymphocyte antigen-4 and thyroid associated ophthalmopathy. Curr eye Res (2015) 40(12):1195–203. 10.3109/02713683.2014.993767 25615025

[15] Li F Ma X Du L Shi L Cao Q Li N. Identification of susceptibility SNPs in CTLA-4 and PTPN22 for scleritis in Han Chinese. Clin Exp Immunol (2019) 197(2):230–6. 10.1111/cei.13298 PMC6642872 30921471

[16] Li Y Hong M Huang X Zhong L Gu Y Wang D. PD-1 Polymorphisms are associated with susceptibility of acute anterior uveitis in Chinese population. DNA Cell Biol (2019) 38(2):121–8. 10.1089/dna.2018.4417 30540488

[17] Meng Q Liu X Yang P Hou S Du L Zhou H. PDCD1 genes may protect against extraocular manifestations in Chinese Han patients with Vogt-Koyanagi-Harada syndrome. Mol Vision (2009) 15:386–92. PMC2645903 19234630

[18] Chen L Pai V Levinson R Sharpe AH Freeman GJ Braun J. Constitutive neuronal expression of the immune regulator, programmed death 1 (PD-1), identified during experimental autoimmune uveitis. Ocular Immunol Inflammation (2009) 17(1):47–55. 10.1080/09273940802491884 19294574

[19] Wang X Wu M Cao Y Zhang Z Guo F Li X. Exploring the role of programmed cell death protein 1 and its ligand 1 in eye diseases. Crit Rev Clin Lab Sci (2019) 56(1):18–32. 10.1080/10408363.2018.1522292 30602320

[20] Chang B Huang T Wei H Shen L Zhu D He W. The correlation and prognostic value of serum levels of soluble programmed death protein 1 (sPD-1) and soluble programmed death-ligand 1 (sPD-L1) in patients with hepatocellular carcinoma. Cancer Immunol Immunother CII (2019) 68(3):353–63. 10.1007/s00262-018-2271-4 PMC6426820 30506460

[21] Purcell S Neale B Todd-Brown K Thomas L Ferreira MA Bender D. PLINK: a tool set for whole-genome association and population-based linkage analyses. Am J Hum Genet (2007) 81(3):559–75. 10.1086/519795 PMC1950838 17701901

[22] Barrett JC Fry B Maller J Daly MJ. Haploview: analysis and visualization of LD and haplotype maps. Bioinf (Oxford England) (2005) 21(2):263–5. 10.1093/bioinformatics/bth457 15297300

[23] Sun T Zhou Y Yang M Hu Z Tan W Han X. Functional genetic variations in cytotoxic T-lymphocyte antigen 4 and susceptibility to multiple types of cancer. Cancer Res (2008) 68(17):7025–34. 10.1158/0008-5472.Can-08-0806 18757416

[24] Mäurer M Loserth S Kolb-Mäurer A Ponath A Wiese S Kruse N. A polymorphism in the human cytotoxic T-lymphocyte antigen 4 (CTLA4) gene (exon 1 +49) alters T-cell activation. Immunogenetics (2002) 54(1):1–8. 10.1007/s00251-002-0429-9 11976786

[25] Wang XB Pirskanen R Giscombe R Lefvert AK. Two SNPs in the promoter region of the CTLA-4 gene affect binding of transcription factors and are associated with human myasthenia gravis. J Internal Med (2008) 263(1):61–9. 10.1111/j.1365-2796.2007.01879.x 18088253

[26] Daroszewski J Pawlak E Karabon L Frydecka I Jonkisz A Slowik M. Soluble CTLA-4 receptor an immunological marker of Graves’ disease and severity of ophthalmopathy is associated with CTLA-4 Jo31 and CT60 gene polymorphisms. Eur J Endocrinol (2009) 161(5):787–93. 10.1530/eje-09-0600 19734241

[27] Wang XB Kakoulidou M Giscombe R Qiu Q Huang D Pirskanen R. Abnormal expression of CTLA-4 by T cells from patients with myasthenia gravis: effect of an AT-rich gene sequence. J Neuroimmunol (2002) 130(1-2):224–32. 10.1016/s0165-5728(02)00228-x 12225905

[28] Toussirot E Saas P Deschamps M Pouthier F Perrot L Perruche S. Increased production of soluble CTLA-4 in patients with spondylarthropathies correlates with disease activity. Arthritis Res Ther (2009) 11(4):R101. 10.1186/ar2747 19570209 PMC2745776

[29] Linsley PS Greene JL Brady W Bajorath J Ledbetter JA Peach R. Human B7-1 (CD80) and B7-2 (CD86) bind with similar avidities but distinct kinetics to CD28 and CTLA-4 receptors. Immunity (1994) 1(9):793–801. 10.1016/s1074-7613(94)80021-9 7534620

[30] Ward FJ Dahal LN Wijesekera SK Abdul-Jawad SK Kaewarpai T Xu H. The soluble isoform of CTLA-4 as a regulator of T-cell responses. Eur J Immunol (2013) 43(5):1274–85. 10.1002/eji.201242529 23400950

[31] Hofmeyer KA Jeon H Zang X. The PD-1/PD-L1 (B7-H1) pathway in chronic infection-induced cytotoxic T lymphocyte exhaustion. J Biomed Biotechnol (2011) 2011:451694. 10.1155/2011/451694 21960736 PMC3180079

[32] La Rosa C Krishnan A Longmate J Martinez J Manchanda P Lacey SF. Programmed death-1 expression in liver transplant recipients as a prognostic indicator of cytomegalovirus disease. J Infect Dis (2008) 197(1):25–33. 10.1086/523652 18171281

[33] Sester U Presser D Dirks J Gärtner BC Köhler H Sester M. PD-1 expression and IL-2 loss of cytomegalovirus- specific T cells correlates with viremia and reversible functional anergy. Am J Transplant (2008) 8(7):1486–97. 10.1111/j.1600-6143.2008.02279.x 18510628

[34] Song MY Park SH Nam HJ Choi DH Sung YC. Enhancement of vaccine-induced primary and memory CD8(+) T-cell responses by soluble PD-1. J Immunother (2011) 34(3):297–306. 10.1097/CJI.0b013e318210ed0e 21389868

[35] Shin SP Seo HH Shin JH Park HB Lim DP Eom HS. Adenovirus expressing both thymidine kinase and soluble PD1 enhances antitumor immunity by strengthening CD8 T-cell response. Mol Ther (2013) 21(3):688–95. 10.1038/mt.2012.252 PMC3589170 23337984

