# Supplementary material for: Polymorphisms and Circulating Plasma Protein Levels of Immune Checkpoints (CTLA-4 and PD-1) Are Associated With Posner-Schlossman Syndrome in Southern Chinese
Source: Front Immunol. 2021 Feb 24;12:607966. doi: 10.3389/fimmu.2021.607966 (PMC7943469; doi:10.3389/fimmu.2021.607966)
Supplement: Supplementary file 1 [file Table_1.docx]

**Supplementary Table 1|** The characteristic information of ten SNPs

| Gene | SNP | Chromosome | Position |  | **Mutation** |
| --- | --- | --- | --- | --- | --- |
| *CTLA-4* | rs733618 | 2:203866221 | upstream |  | T>C |
|  | rs4553808 | 2:203866282 | upstream |  | A>G,T |
|  | rs5742909 | 2:203867624 | upstream |  | C>T |
|  | rs231775 | 2:203867991 | exon1 |  | A>G,T |
|  | rs3087243 | 2:203874196 | downstream |  | G>A |
| *PD-1* | rs10204525 | 2:241850169 | 3’UTR |  | C>T |
|  | rs2227981 | 2:241851121 | exon5 |  | A>C,G |
|  | rs2227982 | 2:241851281 | exon5 |  | G>A |
|  | rs41386349 | 2:241851697 | intron4 |  | G>A,C |
|  | rs36084323 | 2:241859444 | upstream |  | C>T |

SNPs information obtained from GRCh38; SNP: single nucleotide polymorphisins
