# Supplementary material for: Polymorphisms and Circulating Plasma Protein Levels of Immune Checkpoints (CTLA-4 and PD-1) Are Associated With Posner-Schlossman Syndrome in Southern Chinese
Source: Front Immunol. 2021 Feb 24;12:607966. doi: 10.3389/fimmu.2021.607966 (PMC7943469; doi:10.3389/fimmu.2021.607966)
Supplement: Supplementary file 2 [file Table_2.docx]

Supplementary Material

# Supplementary Table 2|

# Product size and primers of the SNPs in *CTLA-4* and *PD-1* genes

| SNP ID | Product size (bp) | PCR primer sequence | Extension primer sequence |
| --- | --- | --- | --- |
| *CTLA-4* |  |  |  |
| rs733618 | 231 | F: TGACTTCCACAGGCTGAACCAC  R: CCAGCTCAAGCGCCAACAAG | SF: TTTTTTTTTTTTTTTTTTTTTCTATCATGATCATGG  GTTTAGCTG |
| rs4553808 | 231 | F: TGACTTCCACAGGCTGAACCAC  R: CCAGCTCAAGCGCCAACAAG | SR: TTTTTTTTTTTTTTTTTTTTTTTTTTTTTTTTTTTTTT  TTTTTTTTTGACTGGGCAACAGAGGTTTTT |
| rs5742909 | 244 | F: TGGGATTTAGGAGGACCCTTGTACT  R: TGCACACACAGAAGGCACTTGA | SF: TTTTTTTTTTTTTAAGTCTCCACTTAGTTATCCAGAT  CCT |
| rs231775 | 178 | F: CCTGAACACCGCTCCCATAAAG  R: GGAGAAACACCTCCTCCATCTTCA | SF: TTTTTTTTTTTTTTTTTTTTTTTTTTTTTTTTTTTTTT  TTTTTTTTTTTTTTTTTTTGGCTCAGCTGAACCTGGCT |
| rs3087243 | 193 | F: GTCAGCTTTGCACCAGCCATTA  R: GAGCTGAGAAAGCAGGCGGTAA | SF: TTTTTTTTTTTTTTATTTCTTCACCACTATTTGGGA  TATAAC |
| *PD-1* |  |  |  |
| rs10204525 | 103 | F: CCGGCCAACCCCTTTAAATAAT  R: GTGTTGGGAGGGCAGAAGTGC | SF: TAGAGCTCCCAGGGTGGGCA |
| rs2227981 | 479 | F: TCTCCTGAGGAAATGCGCTGAC  R: GTGGTGTCCCCAGATCACACAG | SR: TTTTTTTTTTTTTTTTTTTTTTTTTTTTTTTTTTTTTT  TTTTTTTTTTTTTTTTTTCCGCCCGCAGGGGCTCAGC |
| rs2227982 | 479 | F: TCTCCTGAGGAAATGCGCTGAC  R: GTGGTGTCCCCAGATCACACAG | SF: TTTTTTTTTTTTCCACAGAGAACACAGGCACG |
| rs41386349 | 230 | F: AGACCGCAGGCAGGCACATA  R: GGCCTGCAGGACTCACATTCT | SF: TTTTTACCGCAGGCGGGCACACGC |
| rs36084323 | 231 | F: CTCCCATTCTGTCGGAGCCTCT  R: AGGCATCACACGGTGGAAAGAT | SF: TTTTTTTTTTGCCTGGCCTCTGCCTTCC |

F, forward; R, reverse.
