# Supplementary material for: Polymorphisms and Circulating Plasma Protein Levels of Immune Checkpoints (CTLA-4 and PD-1) Are Associated With Posner-Schlossman Syndrome in Southern Chinese
Source: Front Immunol. 2021 Feb 24;12:607966. doi: 10.3389/fimmu.2021.607966 (PMC7943469; doi:10.3389/fimmu.2021.607966)
Supplement: Supplementary file 3 [file Table_3.docx]

Supplementary Material

# Supplementary Table 3|

# The raw genotype data of the samples used in this study

| Sample name | Age | Sex | rs733618 | rs4553808 | rs5742909 | rs231775 | rs3087243 | rs10204525 | rs2227981 | rs2227982 | rs41386349 | rs36084323 |
| --- | --- | --- | --- | --- | --- | --- | --- | --- | --- | --- | --- | --- |
| Patient 1 | 69 | male | T/T | A/A | C/C | G/A | G/A | C/T | A/A | G/G | G/A | C/T |
| Patient 2 | 38 | male | C/C | A/A | C/C | G/G | G/G | C/T | G/G | G/A | G/G | C/T |
| Patient 3 | 57 | female | T/T | A/A | C/C | G/G | G/G | C/T | G/G | G/A | G/G | C/T |
| Patient 4 | 40 | female | T/T | A/A | C/C | G/A | G/A | C/T | G/A | G/A | G/G | C/T |
| Patient 5 | 34 | male | C/C | A/A | C/C | G/G | G/G | C/T | G/G | G/A | G/G | C/T |
| Patient 6 | 38 | female | T/T | A/A | C/C | A/A | A/A | C/T | G/G | G/A | G/G | C/T |
| Patient 7 | 40 | male | T/T | A/A | C/C | G/A | G/A | T/T | G/G | A/A | G/G | T/T |
| Patient 8 | 29 | male | C/T | A/A | C/C | G/G | G/G | C/T | G/G | G/A | G/G | C/T |
| Patient 9 | 29 | female | T/T | A/A | C/C | G/A | G/A | T/T | G/G | A/A | G/G | T/T |
| Patient 10 | 30 | male | C/C | A/A | C/C | G/G | G/G | T/T | G/G | A/A | G/G | T/T |
| Patient 11 | 46 | male | T/T | G/A | C/T | A/A | G/A | T/T | G/A | G/A | G/A | C/T |
| Patient 12 | 49 | male | C/T | A/A | C/C | G/A | G/A | C/T | A/A | G/G | G/A | C/C |
| Patient 13 | 60 | female | C/T | A/A | C/C | G/A | G/A | C/T | G/A | G/G | G/A | C/C |
| Patient 14 | 30 | female | T/T | A/A | C/C | G/A | G/A | C/C | G/G | G/G | G/G | C/C |
| Patient 15 | 32 | female | T/T | A/A | C/C | A/A | A/A | T/T | G/A | G/A | G/A | C/T |
| Patient 16 | 25 | male | C/T | A/A | C/C | G/A | G/A | T/T | G/G | A/A | G/G | T/T |
| Patient 17 | 51 | female | T/T | A/A | C/C | A/A | A/A | T/T | G/G | A/A | G/G | T/T |
| Patient 18 | 48 | male | C/T | A/A | C/C | G/G | G/G | T/T | G/G | A/A | G/G | T/T |
| Patient 19 | 19 | female | C/C | A/A | C/C | G/G | G/G | T/T | G/A | G/A | G/A | C/T |
| Patient 20 | 51 | male | T/T | G/A | C/T | A/A | G/A | C/T | G/A | G/G | G/A | C/C |
| Patient 21 | 71 | female | C/T | A/A | C/C | G/G | G/G | T/T | G/G | A/A | G/G | T/T |
| Patient 22 | 38 | male | T/T | G/A | C/T | G/A | G/G | C/C | A/A | G/G | G/G | C/C |
| Patient 23 | 51 | male | T/T | G/A | C/T | A/A | G/A | T/T | G/G | A/A | G/G | T/T |
| Patient 24 | 39 | female | T/T | A/A | C/C | G/G | G/G | T/T | A/A | G/G | A/A | C/T |
| Patient 25 | 33 | female | C/T | A/A | C/C | G/A | G/A | C/T | G/G | G/A | G/G | C/T |
| Patient 26 | 22 | female | C/T | G/A | C/T | G/A | G/G | C/T | G/G | G/A | G/G | C/T |
| Patient 27 | 26 | female | C/T | A/A | C/C | G/G | G/G | T/T | G/G | A/A | G/G | T/T |
| Patient 28 | 56 | male | T/T | G/A | C/T | G/A | G/G | T/T | G/G | A/A | G/G | T/T |
| Patient 29 | 48 | male | T/T | A/A | C/C | G/G | G/G | T/T | G/G | A/A | G/G | T/T |
| Patient 30 | 35 | female | C/T | A/A | C/C | G/A | G/A | C/T | G/G | G/A | G/G | C/T |
| Patient 31 | 34 | male | C/C | A/A | C/C | G/G | G/G | T/T | G/G | A/A | G/G | T/T |
| Patient 32 | 32 | female | C/T | A/A | C/C | G/A | G/A | C/T | G/G | G/A | G/G | C/T |
| Patient 33 | 52 | male | T/T | A/A | C/C | G/A | G/A | T/T | G/A | G/A | G/A | C/T |
| Patient 34 | 51 | female | C/T | A/A | C/C | G/G | G/G | C/T | G/G | G/A | G/G | C/T |
| Patient 35 | 43 | male | T/T | A/A | C/C | G/A | G/A | T/T | A/A | G/G | A/A | C/C |
| Patient 36 | 29 | male | C/C | A/A | C/C | G/G | G/G | T/T | G/G | A/A | G/G | T/T |
| Patient 37 | 33 | female | C/T | A/A | C/C | G/A | G/A | C/T | A/A | G/G | G/A | C/C |
| Patient 38 | 40 | female | T/T | G/G | T/T | A/A | G/G | T/T | G/G | A/A | G/G | T/T |
| Patient 39 | 38 | male | C/C | A/A | C/C | G/G | G/G | C/T | G/A | G/G | G/A | C/C |
| Patient 40 | 52 | female | T/T | G/A | C/T | G/A | G/G | C/T | G/A | G/A | G/G | C/T |
| Patient 41 | 45 | male | T/T | A/A | C/C | A/A | A/A | C/T | G/A | G/G | G/A | C/C |
| Patient 42 | 19 | male | T/T | A/A | C/C | A/A | A/A | T/T | G/A | G/A | G/A | C/T |
| Patient 43 | 31 | female | T/T | A/A | C/C | G/G | G/G | C/T | G/G | G/A | G/G | C/T |
| Patient 44 | 65 | female | T/T | A/A | C/C | G/A | G/A | C/T | G/G | G/A | G/G | C/T |
| Patient 45 | 52 | female | C/T | A/A | C/C | G/A | G/A | C/T | G/G | G/A | G/G | C/T |
| Patient 46 | 41 | male | T/T | A/A | C/C | G/A | G/A | C/C | G/G | G/G | G/G | C/C |
| Patient 47 | 56 | male | C/T | A/A | C/C | G/A | G/A | C/C | G/G | G/G | G/G | C/C |
| Patient 48 | 34 | female | T/T | A/A | C/C | G/G | G/G | C/T | G/A | G/A | G/G | C/T |
| Patient 49 | 39 | female | C/T | A/A | C/C | G/A | G/A | T/T | G/G | A/A | G/G | T/T |
| Patient 50 | 59 | female | T/T | A/A | C/C | G/A | G/A | T/T | G/G | A/A | G/G | T/T |
| Patient 51 | 28 | male | T/T | A/A | C/C | A/A | A/A | C/T | G/A | G/G | G/A | C/C |
| Patient 52 | 28 | male | C/T | G/A | C/T | G/A | G/G | T/T | G/G | A/A | G/G | T/T |
| Patient 53 | 46 | female | C/T | G/A | C/T | G/A | G/G | C/T | G/G | G/A | G/G | C/C |
| Patient 54 | 32 | male | C/T | A/A | C/C | G/A | G/A | C/T | G/G | G/A | G/G | C/T |
| Patient 55 | 37 | female | T/T | A/A | C/C | G/A | G/A | T/T | G/A | G/A | G/A | C/T |
| Patient 56 | 56 | male | C/T | A/A | C/C | G/G | G/G | T/T | G/A | G/A | G/A | C/T |
| Patient 57 | 22 | female | T/T | G/A | C/T | A/A | G/A | T/T | G/G | A/A | G/G | C/T |
| Patient 58 | 41 | male | C/T | A/A | C/C | G/A | G/A | C/T | G/A | G/G | G/A | C/C |
| Patient 59 | 32 | male | T/T | G/A | C/T | A/A | G/A | T/T | G/G | A/A | G/G | T/T |
| Patient 60 | 35 | female | C/T | G/A | C/T | G/A | G/G | T/T | G/A | G/A | G/A | C/T |
| Patient 61 | 31 | male | T/T | G/A | C/T | G/A | G/G | C/T | A/A | G/G | G/A | C/C |
| Patient 62 | 47 | male | T/T | A/A | C/C | G/G | G/G | T/T | G/G | A/A | G/G | T/T |
| Patient 63 | 37 | female | C/T | A/A | C/C | G/G | G/G | C/T | G/G | G/A | G/G | C/T |
| Patient 64 | 40 | female | T/T | A/A | C/C | G/A | G/A | C/T | G/G | G/A | G/G | C/T |
| Patient 65 | 63 | female | T/T | G/A | C/T | G/A | G/G | C/T | G/G | G/A | G/G | C/T |
| Patient 66 | 31 | male | T/T | A/A | C/C | G/G | G/G | T/T | G/A | G/A | G/A | C/T |
| Patient 67 | 54 | female | C/C | A/A | C/C | G/G | G/G | C/T | G/A | G/A | G/G | C/T |
| Patient 68 | 23 | female | C/T | A/A | C/C | G/A | G/A | C/T | G/G | G/A | G/G | C/T |
| Patient 69 | 30 | female | T/T | A/A | C/C | G/G | G/G | T/T | G/G | A/A | G/G | T/T |
| Patient 70 | 30 | male | C/T | A/A | C/C | G/A | G/A | C/T | G/G | G/A | G/G | C/T |
| Patient 71 | 35 | female | C/T | A/A | C/C | G/G | G/G | T/T | G/A | G/A | G/A | C/T |
| Patient 72 | 69 | male | T/T | A/A | C/C | G/G | G/G | C/T | G/G | G/A | G/G | C/T |
| Patient 73 | 53 | male | T/T | A/A | C/C | A/A | A/A | C/T | G/A | G/G | G/A | C/C |
| Patient 74 | 42 | female | C/T | A/A | C/C | G/G | G/G | T/T | G/G | A/A | G/G | T/T |
| Patient 75 | 33 | male | C/T | A/A | C/C | G/G | G/G | T/T | G/A | G/A | G/A | C/T |
| Patient 76 | 47 | male | C/C | A/A | C/C | G/G | G/G | T/T | G/G | A/A | G/G | T/T |
| Patient 77 | 43 | male | C/T | A/A | C/C | G/G | G/G | T/T | G/G | A/A | G/G | T/T |
| Patient 78 | 60 | male | T/T | G/A | C/T | G/A | G/G | T/T | A/A | G/G | A/A | C/C |
| Patient 79 | 49 | male | C/T | A/A | C/C | G/A | G/A | T/T | A/A | G/G | A/A | C/C |
| Patient 80 | 29 | male | T/T | G/A | C/T | A/A | G/A | T/T | A/A | G/G | A/A | C/C |
| Patient 81 | 26 | male | T/T | A/A | C/C | G/A | G/A | T/T | G/G | A/A | G/G | T/T |
| Patient 82 | 43 | female | C/T | A/A | C/C | G/G | G/G | T/T | G/G | A/A | G/G | T/T |
| Patient 83 | 43 | male | T/T | G/G | T/T | A/A | G/G | T/T | G/A | G/A | G/A | C/T |
| Patient 84 | 30 | male | C/C | A/A | C/C | G/G | G/G | C/T | G/G | G/A | G/G | C/T |
| Patient 85 | 47 | female | C/T | A/A | C/C | G/G | G/G | C/T | G/A | G/G | G/A | C/C |
| Patient 86 | 43 | female | C/T | A/A | C/C | G/A | G/A | C/T | G/G | G/A | G/G | C/T |
| Patient 87 | 66 | male | C/T | A/A | C/C | G/G | G/G | C/T | G/G | G/A | G/G | C/T |
| Patient 88 | 32 | female | T/T | G/A | C/T | G/A | G/G | C/T | G/A | G/G | G/A | C/C |
| Patient 89 | 32 | male | T/T | G/A | C/T | A/A | G/A | C/T | A/A | G/G | G/A | C/C |
| Patient 90 | 36 | female | T/T | G/G | T/T | A/A | G/G | C/C | G/G | G/G | G/G | C/C |
| Patient 91 | 40 | male | T/T | G/A | C/T | G/A | G/G | T/T | A/A | G/G | A/A | C/C |
| Patient 92 | 30 | male | T/T | G/A | C/C | A/A | G/A | T/T | G/G | A/A | G/G | T/T |
| Patient 93 | 33 | male | C/T | A/A | C/C | G/A | G/A | T/T | G/A | G/A | G/A | C/T |
| Patient 94 | 32 | female | T/T | G/A | C/T | A/A | G/A | C/T | G/G | G/A | G/G | C/T |
| Patient 95 | 40 | female | C/T | A/A | C/C | G/A | G/A | C/C | G/A | G/G | G/G | C/C |
| Patient 96 | 26 | female | T/T | A/A | C/C | G/A | G/A | T/T | G/A | G/A | G/A | C/T |
| Patient 97 | 39 | female | C/T | A/A | C/C | G/G | G/G | C/T | G/A | G/G | G/A | C/C |
| Patient 98 | 47 | female | C/T | A/A | C/C | G/G | G/G | C/T | G/G | G/A | G/G | C/T |
| Patient 99 | 39 | female | C/C | A/A | C/C | G/G | G/G | T/T | G/G | A/A | G/G | T/T |
| Patient 100 | 45 | male | C/T | A/A | C/C | G/G | G/G | C/T | G/G | G/A | G/G | C/T |
| Patient 101 | 31 | female | T/T | A/A | C/C | G/A | G/A | C/T | G/A | G/G | G/A | C/C |
| Patient 102 | 42 | male | T/T | A/A | C/C | G/G | G/G | T/T | G/G | A/A | G/G | T/T |
| Patient 103 | 34 | female | C/C | A/A | C/C | G/G | G/G | C/T | A/A | G/G | G/A | C/C |
| Patient 104 | 25 | male | T/T | G/A | C/T | G/A | G/G | C/T | G/A | G/A | G/G | C/T |
| Patient 105 | 19 | male | T/T | A/A | C/C | G/A | G/A | C/T | G/A | G/A | G/G | C/T |
| Patient 106 | 51 | male | T/T | G/G | T/T | A/A | G/G | C/T | G/A | G/A | G/G | C/T |
| Patient 107 | 32 | male | C/T | A/A | C/C | G/G | G/G | C/T | G/A | G/A | G/G | C/T |
| Patient 108 | 42 | male | T/T | A/A | C/C | A/A | A/A | T/T | G/A | G/A | G/A | C/T |
| Patient 109 | 50 | male | T/T | A/A | C/C | G/G | G/G | C/T | G/A | G/G | G/A | C/C |
| Patient 110 | 26 | female | T/T | A/A | C/C | A/A | A/A | T/T | G/A | G/A | G/A | C/T |
| Patient 111 | 31 | male | T/T | A/A | C/C | G/G | G/G | T/T | A/A | G/G | A/A | C/C |
| Patient 112 | 42 | female | C/T | A/A | C/C | G/G | G/G | C/T | G/A | G/A | G/G | C/T |
| Patient 113 | 46 | female | C/T | G/A | C/T | G/A | G/G | T/T | G/G | A/A | G/G | T/T |
| Patient 114 | 41 | male | C/T | A/A | C/C | G/G | G/G | C/T | G/A | G/A | G/G | C/T |
| Patient 115 | 40 | male | C/T | A/A | C/C | G/G | G/G | C/C | G/A | G/G | G/G | C/C |
| Patient 116 | 36 | male | T/T | A/A | C/C | A/A | A/A | T/T | G/A | G/A | G/A | C/T |
| Patient 117 | 42 | male | C/C | A/A | C/C | G/G | G/G | T/T | G/G | A/A | G/G | T/T |
| Patient 118 | 46 | female | C/T | A/A | C/C | G/G | G/G | T/T | G/G | A/A | G/G | T/T |
| Patient 119 | 45 | female | T/T | A/A | C/C | A/A | A/A | T/T | A/A | G/G | A/A | C/C |
| Patient 120 | 36 | male | C/C | A/A | C/C | G/G | G/G | T/T | G/A | G/A | G/A | C/T |
| Patient 121 | 37 | female | T/T | A/A | C/C | G/G | G/G | T/T | G/A | G/A | G/A | C/T |
| Patient 122 | 23 | male | C/T | A/A | C/C | G/G | G/G | T/T | G/G | A/A | G/G | T/T |
| Patient 123 | 36 | female | T/T | G/A | C/T | G/A | G/G | C/C | G/G | G/G | G/G | C/C |
| Patient 124 | 39 | male | T/T | G/A | C/T | A/A | G/A | T/T | G/G | A/A | G/G | T/T |
| Patient 125 | 28 | male | T/T | G/G | T/T | A/A | G/G | T/T | G/G | A/A | G/G | T/T |
| Patient 126 | 24 | female | T/T | A/A | C/C | A/A | A/A | T/T | A/A | G/G | A/A | C/C |
| Patient 127 | 57 | female | C/T | A/A | C/C | G/G | G/G | T/T | G/G | A/A | G/G | T/T |
| Patient 128 | 39 | male | T/T | G/A | C/T | A/A | G/A | T/T | G/G | A/A | G/G | T/T |
| Patient 129 | 23 | female | T/T | G/G | T/T | A/A | G/G | T/T | G/G | A/A | G/G | T/T |
| Patient 130 | 33 | male | C/T | A/A | C/C | G/G | G/G | C/T | G/A | G/G | G/A | C/C |
| Patient 131 | 76 | male | T/T | A/A | C/C | G/A | G/A | C/T | G/G | G/A | G/G | C/T |
| Patient 132 | 34 | male | C/T | A/A | C/C | G/G | G/G | T/T | A/A | G/G | A/A | C/C |
| Patient 133 | 31 | female | C/T | G/A | C/T | G/A | G/G | T/T | G/G | A/A | G/G | T/T |
| Patient 134 | 34 | female | T/T | A/A | C/C | G/G | G/G | T/T | G/G | A/A | G/G | T/T |
| Patient 135 | 70 | female | C/T | A/A | C/C | G/G | G/G | T/T | G/A | G/A | G/A | C/T |
| Patient 136 | 35 | female | T/T | G/A | C/T | A/A | G/A | C/T | G/G | G/A | G/G | C/T |
| Patient 137 | 42 | male | C/T | A/A | C/C | G/A | G/A | C/T | G/G | G/A | G/G | C/T |
| Control 1 | 52 | male | T/T | A/A | C/C | G/G | G/G | T/T | G/G | A/A | G/G | T/T |
| Control 2 | 42 | male | T/T | G/A | C/T | G/A | G/G | T/T | G/A | G/A | G/A | T/T |
| Control 3 | 40 | male | C/T | A/A | C/C | G/A | G/A | T/T | G/G | A/A | G/G | T/T |
| Control 4 | 23 | female | T/T | G/A | C/T | G/A | G/G | C/T | G/G | G/A | G/G | C/T |
| Control 5 | 38 | male | T/T | G/A | C/T | G/A | G/G | T/T | G/A | G/A | G/A | C/T |
| Control 6 | 45 | male | C/C | A/A | C/C | G/G | G/G | T/T | G/G | A/A | G/G | C/T |
| Control 7 | 33 | female | C/T | G/A | C/T | G/A | G/G | T/T | G/G | A/A | G/G | T/T |
| Control 8 | 55 | female | T/T | G/A | C/T | G/A | G/G | T/T | G/G | A/A | G/G | C/T |
| Control 9 | 23 | male | C/T | A/A | C/C | G/G | G/G | T/T | G/G | A/A | G/G | T/T |
| Control 10 | 46 | male | C/C | A/A | C/C | G/G | G/G | C/C | G/G | G/G | G/G | C/C |
| Control 11 | 34 | male | T/T | G/A | C/T | G/A | G/G | T/T | G/G | A/A | G/G | T/T |
| Control 12 | 47 | female | T/T | A/A | C/C | A/A | A/A | C/T | G/A | G/G | G/A | C/C |
| Control 13 | 31 | male | C/C | A/A | C/C | G/G | G/G | T/T | G/A | G/A | G/A | C/T |
| Control 14 | 25 | male | C/C | A/A | C/C | G/G | G/G | T/T | G/G | A/A | G/G | T/T |
| Control 15 | 50 | male | C/T | A/A | C/C | G/G | G/G | T/T | G/G | A/A | G/G | T/T |
| Control 16 | 41 | female | T/T | G/A | C/T | G/A | G/G | T/T | G/G | A/A | G/G | T/T |
| Control 17 | 40 | male | C/T | A/A | C/C | G/G | G/G | C/T | G/G | G/A | G/G | C/T |
| Control 18 | 52 | male | C/T | A/A | C/C | G/G | G/G | C/T | A/A | G/G | G/A | C/C |
| Control 19 | 47 | male | C/T | A/A | C/C | G/G | G/G | C/T | A/A | G/G | G/A | C/C |
| Control 20 | 51 | male | T/T | A/A | C/C | G/A | G/A | C/T | G/A | G/G | G/A | C/C |
| Control 21 | 22 | male | C/T | A/A | C/C | G/G | G/G | T/T | G/A | G/A | G/A | C/T |
| Control 22 | 33 | male | C/C | A/A | C/C | G/G | G/G | T/T | G/A | G/A | G/A | C/T |
| Control 23 | 53 | male | T/T | G/A | C/T | A/A | G/A | T/T | G/G | A/A | G/G | T/T |
| Control 24 | 52 | male | T/T | A/A | C/C | G/G | G/G | T/T | G/A | G/A | G/A | C/C |
| Control 25 | 35 | male | T/T | G/A | C/T | A/A | G/A | T/T | G/G | A/A | G/G | T/T |
| Control 26 | 31 | female | C/T | A/A | C/C | G/G | G/G | T/T | G/A | G/A | G/A | C/T |
| Control 27 | 43 | male | C/T | A/A | C/C | G/G | G/G | T/T | G/A | G/A | G/A | C/T |
| Control 28 | 47 | male | T/T | A/A | C/C | G/A | G/A | T/T | G/A | G/A | G/A | C/T |
| Control 29 | 56 | male | C/T | A/A | C/C | G/G | G/G | C/T | G/G | G/A | G/G | C/T |
| Control 30 | 23 | male | T/T | A/A | C/C | G/A | G/A | T/T | G/G | A/A | G/G | T/T |
| Control 31 | 44 | male | C/T | A/A | C/C | G/A | G/A | T/T | G/G | A/A | G/G | T/T |
| Control 32 | 51 | male | T/T | A/A | C/C | G/G | G/G | T/T | G/G | A/A | G/G | T/T |
| Control 33 | 51 | male | T/T | G/A | C/C | G/A | G/A | T/T | G/A | G/A | G/A | C/T |
| Control 34 | 25 | male | C/T | A/A | C/C | G/G | G/G | T/T | G/G | A/A | G/G | T/T |
| Control 35 | 44 | male | C/T | A/A | C/C | G/G | G/G | C/T | A/A | G/G | G/A | C/C |
| Control 36 | 34 | male | C/T | A/A | C/C | G/A | G/A | T/T | A/A | G/G | A/A | C/C |
| Control 37 | 44 | male | C/C | A/A | C/C | G/G | G/G | T/T | A/A | G/G | A/A | C/C |
| Control 38 | 48 | male | C/T | A/A | C/C | G/A | G/A | T/T | G/G | A/A | G/G | T/T |
| Control 39 | 36 | male | C/T | A/A | C/C | G/A | G/A | T/T | G/A | G/A | G/A | C/T |
| Control 40 | 46 | male | C/T | A/A | C/C | G/G | G/G | C/C | G/A | G/G | G/G | C/C |
| Control 41 | 45 | male | T/T | A/A | C/C | G/A | G/A | C/C | G/G | G/G | G/G | C/C |
| Control 42 | 40 | male | C/C | A/A | C/C | G/G | G/G | T/T | G/A | G/A | G/A | T/T |
| Control 43 | 35 | male | T/T | A/A | C/C | G/G | G/G | C/T | G/G | G/A | G/G | C/T |
| Control 44 | 34 | male | C/T | A/A | C/C | G/G | G/G | T/T | G/A | G/A | G/A | C/T |
| Control 45 | 58 | male | T/T | G/A | C/T | A/A | G/A | T/T | G/A | G/A | G/A | C/T |
| Control 46 | 18 | male | C/T | A/A | C/C | G/G | G/G | C/T | G/A | G/G | G/A | C/C |
| Control 47 | 43 | male | C/C | A/A | C/C | G/G | G/G | C/T | G/G | G/A | G/G | C/T |
| Control 48 | 49 | male | C/T | A/A | C/C | G/G | G/G | C/T | G/A | G/G | G/A | C/C |
| Control 49 | 41 | male | T/T | A/A | C/C | G/G | G/G | C/T | G/G | G/A | G/G | C/T |
| Control 50 | 48 | male | C/T | A/A | C/C | G/G | G/G | C/T | G/G | G/A | G/G | C/T |
| Control 51 | 47 | male | T/T | A/A | C/C | G/A | G/A | T/T | G/A | G/A | G/A | C/T |
| Control 52 | 28 | male | T/T | A/A | C/C | G/A | G/A | C/T | G/G | G/A | G/G | C/T |
| Control 53 | 29 | male | T/T | A/A | C/C | G/G | G/G | C/T | G/G | G/A | G/G | C/T |
| Control 54 | 61 | male | T/T | A/A | C/C | G/G | G/G | C/T | G/A | G/G | G/A | C/T |
| Control 55 | 50 | male | C/T | A/A | C/C | G/A | G/A | T/T | G/G | A/A | G/G | T/T |
| Control 56 | 30 | male | C/C | A/A | C/C | G/G | G/G | T/T | G/G | A/A | G/G | T/T |
| Control 57 | 51 | male | C/C | A/A | C/C | G/G | G/G | C/T | G/G | G/A | G/G | C/T |
| Control 58 | 56 | male | C/C | A/A | C/C | G/G | G/G | T/T | G/G | A/A | G/G | C/T |
| Control 59 | 26 | female | C/T | G/A | C/T | G/A | G/G | T/T | G/A | G/A | G/A | C/T |
| Control 60 | 40 | female | C/T | A/A | C/C | G/G | G/G | C/T | G/G | G/A | G/G | C/T |
| Control 61 | 47 | female | C/T | A/A | C/C | G/A | G/A | T/T | G/A | G/A | G/A | C/T |
| Control 62 | 22 | female | C/T | A/A | C/C | G/A | G/A | T/T | A/A | G/G | A/A | C/C |
| Control 63 | 25 | female | C/C | A/A | C/C | G/G | G/G | T/T | G/G | A/A | G/G | T/T |
| Control 64 | 40 | female | C/C | A/A | C/C | G/G | G/G | C/T | G/A | G/G | G/A | C/C |
| Control 65 | 42 | female | C/T | A/A | C/C | G/A | G/A | C/T | G/A | G/A | G/G | C/T |
| Control 66 | 41 | female | C/T | A/A | C/C | G/A | G/A | C/T | G/G | G/A | G/G | C/T |
| Control 67 | 34 | female | C/T | G/A | C/T | G/A | G/G | T/T | G/G | A/A | G/G | T/T |
| Control 68 | 36 | female | C/T | G/A | C/T | G/A | G/G | T/T | G/A | G/A | G/A | C/T |
| Control 69 | 39 | female | T/T | G/A | C/T | G/A | G/G | T/T | A/A | G/G | A/A | C/C |
| Control 70 | 29 | female | C/T | A/A | C/C | G/A | G/A | T/T | G/A | G/A | G/A | C/T |
| Control 71 | 52 | female | C/C | A/A | C/C | G/G | G/G | C/T | G/G | G/A | G/G | C/T |
| Control 72 | 20 | female | C/T | G/A | C/T | G/A | G/G | C/T | G/G | G/A | G/G | C/T |
| Control 73 | 46 | female | C/T | G/A | C/T | G/A | G/G | T/T | G/G | A/A | G/G | T/T |
| Control 74 | 44 | female | T/T | G/A | C/T | A/A | G/A | T/T | G/A | G/A | G/A | C/T |
| Control 75 | 48 | male | C/T | A/A | C/C | G/G | G/G | T/T | G/G | A/A | G/G | T/T |
| Control 76 | 43 | male | T/T | A/A | C/C | G/A | G/A | T/T | G/A | G/A | G/A | C/T |
| Control 77 | 45 | male | C/T | A/A | C/C | G/A | G/A | T/T | G/G | A/A | G/G | T/T |
| Control 78 | 26 | male | T/T | A/A | C/C | G/G | G/G | C/C | G/A | G/G | G/G | C/C |
| Control 79 | 57 | male | T/T | A/A | C/C | G/A | G/A | T/T | G/G | A/A | G/G | T/T |
| Control 80 | 45 | male | C/C | A/A | C/C | G/G | G/G | T/T | G/G | A/A | G/G | T/T |
| Control 81 | 29 | male | C/T | A/A | C/C | G/G | G/G | T/T | A/A | G/G | A/A | C/C |
| Control 82 | 49 | male | T/T | A/A | C/C | G/A | G/A | T/T | G/A | G/A | G/A | C/T |
| Control 83 | 49 | male | C/T | A/A | C/C | G/G | G/G | C/T | G/A | G/A | G/G | C/T |
| Control 84 | 39 | male | C/T | A/A | C/C | G/G | G/G | C/T | G/G | G/A | G/G | C/T |
| Control 85 | 28 | male | C/T | A/A | C/C | G/A | G/A | T/T | G/A | G/A | G/A | C/T |
| Control 86 | 49 | male | C/T | A/A | C/C | G/A | G/A | T/T | G/G | A/A | G/G | T/T |
| Control 87 | 51 | male | C/C | A/A | C/C | G/G | G/G | C/C | G/G | G/G | G/G | C/C |
| Control 88 | 42 | male | T/T | A/A | C/C | G/G | G/G | T/T | G/A | G/A | G/A | C/T |
| Control 89 | 35 | male | T/T | G/A | C/T | A/A | G/A | C/T | G/A | G/A | G/G | C/T |
| Control 90 | 48 | male | T/T | A/A | C/C | G/G | G/G | T/T | G/G | A/A | G/G | T/T |
| Control 91 | 50 | male | C/C | A/A | C/C | G/G | G/G | C/T | G/G | G/A | G/G | C/T |
| Control 92 | 41 | male | T/T | G/A | C/T | G/A | G/G | C/T | G/G | G/A | G/G | C/T |
| Control 93 | 45 | male | C/T | A/A | C/C | G/A | G/A | T/T | G/G | A/A | G/G | T/T |
| Control 94 | 30 | male | C/T | A/A | C/C | G/A | G/A | C/T | G/A | G/G | G/A | C/C |
| Control 95 | 36 | male | T/T | A/A | C/C | G/G | G/G | T/T | G/A | G/A | G/A | C/T |
| Control 96 | 48 | male | C/T | A/A | C/C | G/G | G/G | C/T | G/A | G/A | G/G | C/T |
| Control 97 | 27 | female | T/T | G/G | T/T | A/A | G/G | T/T | G/A | G/A | G/A | C/T |
| Control 98 | 40 | female | C/T | A/A | C/C | G/A | G/A | T/T | G/G | A/A | G/G | T/T |
| Control 99 | 48 | female | C/T | A/A | C/C | G/A | G/A | T/T | G/G | A/A | G/G | C/C |
| Control 100 | 43 | female | T/T | A/A | C/C | G/G | G/G | T/T | G/G | A/A | G/G | T/T |
| Control 101 | 53 | female | C/T | A/A | C/C | G/G | G/G | T/T | G/G | A/A | G/G | C/C |
| Control 102 | 52 | female | C/T | A/A | C/C | G/A | G/A | T/T | G/A | G/A | G/A | C/T |
| Control 103 | 26 | female | C/C | A/A | C/C | G/G | G/G | T/T | G/A | G/A | G/A | C/T |
| Control 104 | 27 | female | T/T | G/A | C/T | G/A | G/G | C/T | G/A | G/A | G/A | C/C |
| Control 105 | 39 | female | C/T | A/A | C/C | G/G | G/G | C/T | G/G | G/A | G/G | C/T |
| Control 106 | 51 | female | T/T | A/A | C/C | G/G | G/G | C/T | G/G | G/A | A/A | C/C |
| Control 107 | 25 | female | C/T | A/A | C/C | G/G | G/G | C/T | G/G | G/A | G/G | C/T |
| Control 108 | 52 | female | C/T | G/A | C/T | G/A | G/G | T/T | G/G | A/A | G/G | T/T |
| Control 109 | 33 | female | C/T | A/A | C/C | G/G | G/G | C/T | G/G | G/A | G/G | C/T |
| Control 110 | 28 | female | T/T | A/A | C/C | G/A | G/A | T/T | G/G | A/A | G/G | T/T |
| Control 111 | 56 | female | T/T | A/A | C/C | G/A | G/A | T/T | G/G | A/A | G/G | C/T |
| Control 112 | 51 | female | C/T | A/A | C/C | G/G | G/G | C/T | G/G | G/A | G/G | C/T |
| Control 113 | 33 | female | C/T | A/A | C/C | G/G | G/G | C/T | G/G | G/A | G/G | C/T |
| Control 114 | 47 | female | C/T | A/A | C/C | G/A | G/A | T/T | G/G | A/A | G/G | T/T |
| Control 115 | 64 | female | C/C | A/A | C/C | G/G | G/G | C/T | G/A | G/G | G/A | C/T |
| Control 116 | 29 | female | C/T | A/A | C/C | G/G | G/G | C/T | G/A | G/G | G/A | C/C |
| Control 117 | 52 | female | T/T | A/A | C/C | A/A | A/A | C/T | G/A | G/G | G/A | C/C |
| Control 118 | 54 | female | C/C | A/A | C/C | G/G | G/G | T/T | G/G | A/A | G/G | T/T |
| Control 119 | 24 | female | C/T | G/A | C/T | G/A | G/G | C/T | G/G | G/A | G/G | C/T |
| Control 120 | 52 | female | C/C | A/A | C/C | G/G | G/G | T/T | G/G | A/A | G/G | T/T |
| Control 121 | 25 | female | C/T | A/A | C/C | G/A | G/A | C/T | A/A | G/G | G/A | C/C |
| Control 122 | 48 | female | C/T | A/A | C/C | G/G | G/G | C/T | G/A | G/G | G/A | C/C |
| Control 123 | 57 | female | C/T | A/A | C/C | G/A | G/A | C/T | G/A | G/A | G/G | C/T |
| Control 124 | 42 | female | C/T | A/A | C/C | G/A | G/A | C/T | G/G | G/A | G/G | C/T |
| Control 125 | 34 | female | C/T | A/A | C/C | G/A | G/A | T/T | G/A | G/A | G/A | C/T |
| Control 126 | 43 | female | T/T | G/G | T/T | A/A | G/G | T/T | G/A | G/A | G/A | C/T |
| Control 127 | 40 | female | T/T | G/A | C/T | A/A | G/A | C/T | G/A | G/A | G/G | C/T |
| Control 128 | 43 | female | C/T | A/A | C/C | G/A | G/A | C/T | G/G | G/A | G/G | C/T |
| Control 129 | 50 | female | C/C | A/A | C/C | G/G | G/G | T/T | G/G | A/A | G/G | T/T |
| Control 130 | 48 | female | C/C | A/A | C/C | G/G | G/G | C/T | G/G | G/A | G/G | C/T |
| Control 131 | 45 | female | C/C | A/A | C/C | G/G | G/G | T/T | G/G | A/A | G/G | T/T |
| Control 132 | 40 | female | C/T | A/A | C/C | G/A | G/A | T/T | G/G | A/A | G/G | T/T |
| Control 133 | 38 | female | T/T | A/A | C/C | A/A | A/A | T/T | G/A | G/A | G/A | T/T |
| Control 134 | 64 | female | C/T | A/A | C/C | G/A | G/A | T/T | G/G | A/A | G/G | T/T |
| Control 135 | 47 | female | T/T | A/A | C/C | A/A | A/A | T/T | G/G | A/A | G/G | T/T |
| Control 136 | 44 | female | T/T | A/A | C/C | G/G | G/G | T/T | G/G | A/A | G/G | T/T |
| Control 137 | 21 | female | T/T | A/A | C/C | G/A | G/A | C/T | G/A | G/G | G/A | C/C |
| Control 138 | 33 | female | T/T | G/A | C/T | A/A | G/A | T/T | G/G | A/A | G/G | T/T |
| Control 139 | 56 | female | C/T | A/A | C/C | G/A | G/A | C/C | G/G | G/G | G/G | C/C |
